# Temporal trend of mortality by suicide among adults in Brazil: 2000 to 2015

**DOI:** 10.47626/2237-6089-2020-0009

**Published:** 2021-02-26

**Authors:** Sasckia Kadishari Medeiros Duarte, Danúbia Hillesheim, Ana Luiza de Lima Curi Hallal

**Affiliations:** 1 Universidade Federal de Santa Catarina FlorianópolisSC Brazil Curso de Graduação em Medicina, Universidade Federal de Santa Catarina (UFSC), Florianópolis , SC , Brazil .; 2 Programa de Pós-graduação em Saúde Coletiva UFSC FlorianópolisSC Brazil Programa de Pós-graduação em Saúde Coletiva , UFSC , Florianópolis , SC , Brazil .; 3 Departamento de Saúde Pública UFSC FlorianópolisSC Brazil Departamento de Saúde Pública , UFSC , Florianópolis , SC , Brazil .

**Keywords:** Epidemiology, suicide, community mental health, adult, regression analysis

## Abstract

**Objective:**

To analyze temporal trends of mortality due to suicide among adults in Brazil, by macroregion and gender, from 2000 to 2015.

**Methods:**

A retrospective study of temporal trends in suicide mortality rates in adults aged 20 to 64 years, by macroregion and gender, from 2000 to 2015. Data from the Brazilian Mortality Database (SIM) and from the Brazilian Institute of Geography and Statistics (IBGE) were used. The mortality rate trends analysis was performed using simple linear regression, with Stata 14 software.

**Results:**

There was an upward trend in mortality due to intentionally inflicted self-harm in the Brazilian adult population in the North, Northeast, and Southeast regions for both genders (p<0.001), with predominance in the male population in these three regions and throughout the country (p<0.001). A downward trend was observed in the South and Midwest (p=0.003 and p=0.040).

**Conclusion:**

Mortality due to intentionally inflicted self-harm has increased in Brazil, but has undergone important variations in different parts of the country. Even a regional analysis is insufficient to achieve a thorough evaluation of these contrasts because of the country’s continental proportions and data collection biases. Further studies focused on this topic are required.

## Introduction

The World Health Organization (WHO) considers suicide a complex phenomenon, with avoidable and multifactorial causes and which affects not only the individual, but also his/her family and the whole community of which he/she is a member. It is the second cause of death in the population of adults aged between 15 and 29 years worldwide and accounts for about 800,000 deaths a year (the equivalent to about 1 person every 40 seconds). ^1^ This number is known to be underestimated due to biases in data collection, which is not performed in certain regions and varies over the years, and also to the pervasive confounding factor of suicide notified as accident or death of indeterminate cause. ^1^

In Brazil, suicide mortality is approximately 11,000 people a year and is the fourth cause of death in the general population (in the age range from 15 to 29 years it is the third cause among men and the eighth among women). ^2^ It has been estimated that the average suicide mortality rate in the country from 1980 to 2000 was 3 to 4 out of every 100,000 inhabitants, which is considered low compared with international standards. In the 1990s, the annual average rose to levels between 5 and 15 per 100,000 inhabitants, which is classified as medium incidence. These numbers change significantly by region, microregion, and under different socioeconomic and climatic conditions. ^3^ However, evidence indicates that suicide rates continue to increase in Brazil, especially among young men and middle-aged women. ^4^

In the last century, suicide was recognized as a subject of interest and a public health problem on a global scale. ^5^ The number of studies on the subject increased as well as researchers’ interest in carrying them out, although the number remains low, with flaws affecting data collection, and the financial resources available are insufficient. The main risk factors for suicide already identified in other studies are: mental diseases and disorders; use of certain medications, drugs and alcohol, exposure to pesticides; terminal and degenerative diseases; and influence of media. ^4 - 7^ Additionally, Brazil has one of the world’s highest levels of income inequality, which has a direct impact on population health and on causes of death. ^8^

There are several studies in the literature describing causes, “suicidogenic” factors, and major influences, both in Brazil and in other parts of the world. ^9 - 13^ In these studies, the main objects of study are risk and protection factors and the characteristics of the populations involved in the act. However, there is a lack of studies evaluating temporal trends in Brazil by macroregions or microregions, mainly more recent ones, or their associations with local conditions. Suicide itself is a sensitive topic, and collection and analysis of data relating to it is difficult and controversial. Furthermore, setting parameters for a country with such large dimensions as Brazil is a challenge.

Considering the above, this study aims to analyze temporal trends in mortality due to suicide in adults in Brazil, by macroregion and gender, from 2000 to 2015.

## Methods

This is a retrospective study of temporal trends in suicide mortality rates in the adult Brazilian population aged between 20 and 64 years, by gender and macroregions, from 2000 to 2015.

In this study, suicides were defined as deaths caused by intentionally inflicted self-harm, codes X60 to X84 from the tenth revision of the International Classification of Diseases (ICD). Data on intentionally inflicted self-harm were obtained from the Brazilian Mortality Database (SIM), maintained by the Ministry of Health (MS) and available via its DATASUS portal. Information on the country’s population and of its regions was obtained from the 2010 national census compiled by the Brazilian Institute of Geography and Statistics (IBGE), together with their respective estimates for the subsequent years.

For data description and analysis, rates of death from intentionally inflicted self-harm were obtained for each calendar year, by macroregion and gender, excluding data without information on gender. Death rates were calculated on Microsoft® Excel® 2016 spreadsheets. Rates were calculated per 100,000 inhabitants.

The analysis of mortality rate trends was conducted using simple linear regression with STATA 14 software. This model was chosen for its straightforward preparation and interpretation and its statistical power. The death rate from intentionally inflicted self-harm was defined as the dependent variable and the years analyzed in the study (2000-2015) were defined as the independent variable. The linear trend was only considered statistically significant when its probability of occurrence was equal to or lower than 0.05, that is, p ≤ 5%.

This study analyzed aggregated public data without identifying the people involved and approval by a Human Research Ethics Committees (HREC) was therefore not required. This study is in compliance with CNS resolution 510, of April 7, 2016.

## Results

From 2000 to 2015, 117,322 adult deaths from intentionally inflicted self-harm occurred in Brazil, which corresponds to 1.72% of all deaths in this period. This sample was 79.7% male and the ratio of about 5 male deaths to 1 female death holds not only in the overall sum of obituaries, but also in the annual distribution in almost all of the years analyzed ( Table 1 ).

**Table 1:** Number of deaths by suicide at the age of 20 to 64 years, according to gender and year of death, Brazil, 2000 to 2015

Year of death	Male	Female	Total
2000	4,434	1,024	5,458
2001	4,993	1,155	6,148
2002	4,935	1,287	6,222
2003	5,118	1,215	6,333
2004	5,161	1,298	6,459
2005	5,521	1,400	6,921
2006	5,641	1,408	7,049
2007	5,710	1,496	7,206
2008	6,094	1,528	7,622
2009	6,215	1,511	7,726
2010	6,041	1,656	7,697
2011	6,369	1,654	8,023
2012	6,543	1,797	8,340
2013	6,798	1,752	8,550
2014	6,873	1,745	8,618
2015	7,071	1,879	8,950
Total	93,517	23,805	117,322

Source: Mortality Information System.

The method most frequently used by the adult population of both genders was hanging or strangulation (X70), which accounted for 59.5% of the total mortality, followed by intentional firearm injury (X71 to X74), used by 8.6%, and self-poisoning using pesticides (X68) and other chemicals or unspecified harmful substances (X69). After hanging or strangulation, the male population preferred firearms (X72 to X74) and pesticides (X 68), while the female population’s second option was pesticides (X 68), followed by other chemicals and harmful substances (X69).

In 2000, there were 5.84 suicide deaths for each 100,000 inhabitants of both genders in Brazil. In 2015, this rate was 7.23. This represents a 19% increase in the incidence of self-harm deaths over the period analyzed. The magnitudes of the male rates are remarkable for all years and are from 3 to 5 times greater than the female rates ( Table 2 ).

**Table 2:** Death rates in adults aged 20 to 64 years, by gender, Brazilian macroregions, and year of death

Region	2000	2001	2002	2003	2004	2005	2006	2007	2008	2009	2010	2011	2012	2013	2014	2015
North																
Male	6.79	7.76	6.28	7.27	7.44	7.64	7.54	8.19	9.88	8.61	8.64	9.65	10.15	10.31	8.70	11.46
Female	1.98	2.09	2.29	1.83	1.85	1.93	2.03	2.04	1.76	1.90	2.35	2.45	1.84	2.17	2.11	2.46
Both	4.46	5.01	4.34	4.62	4.72	4.85	4.84	5.19	5.90	5.32	5.55	6.11	6.07	6.30	5.46	7.02
Northeast																
Male	5.91	7.74	7.50	7.94	8.00	8.88	9.33	9.90	9.73	9.39	8.95	9.90	9.59	10.28	9.69	10.05
Female	1.42	1.69	2.22	2.05	1.89	2.27	1.97	2.31	2.38	2.19	2.29	2.14	2.37	2.22	2.23	2.35
Both	3.62	4.65	4.81	4.94	4.88	5.51	5.57	6.03	5.98	5.71	5.55	5.93	5.89	6.15	5.87	6.10
Southeast																
Male	8.28	9.15	8.95	9.09	8.80	9.18	9.36	8.86	9.47	9.85	10.02	10.29	10.20	10.20	11.02	10.63
Female	1.85	2.18	2.23	2.13	2.18	2.38	2.51	2.46	2.45	2.55	2.71	2.75	2.93	2.79	2.77	2.90
Both	5.01	5.61	5.54	5.56	5.44	5.72	5.88	5.61	5.90	6.15	6.31	6.47	6.51	6.44	6.85	6.72
South																
Male	18.80	19.44	18.11	17.83	17.95	18.70	17.48	17.31	17.53	18.38	15.96	15.45	16.41	16.65	16.16	16.87
Female	3.95	3.84	3.82	3.71	4.13	4.00	3.95	4.19	4.30	3.78	4.05	4.22	4.66	4.33	4.08	4.64
Both	11.29	11.56	10.89	10.70	10.97	11.27	10.65	10.69	10.85	11.01	9.95	9.78	10.48	10.44	10.07	10.71
Midwest																
Male	14.10	13.20	14.65	13.71	12.87	12.35	12.45	12.09	13.85	12.73	11.54	11.97	12.92	13.39	12.99	11.99
Female	3.30	3.41	3.91	3.01	3.87	3.37	3.20	3.25	3.21	3.14	3.64	2.90	3.39	3.55	3.48	3.26
Both	8.70	8.30	9.26	8.34	8.35	7.83	7.80	7.64	8.49	7.90	7.56	7.40	8.11	8.43	8.19	7.59
Brazil																
Male	9.59	10.55	10.19	10.32	10.17	10.65	10.66	10.59	11.09	11.12	10.63	11.03	11.16	11.43	11.40	11.57
Female	2.16	2.38	2.59	2.39	2.50	2.64	2.60	2.71	2.71	2.64	2.84	2.79	2.99	2.87	2.82	3.00
Both	5.84	6.42	6.34	6.31	6.29	6.59	6.58	6.60	6.85	6.82	6.69	6.86	7.02	7.10	7.06	7.23

**Figure 1:**
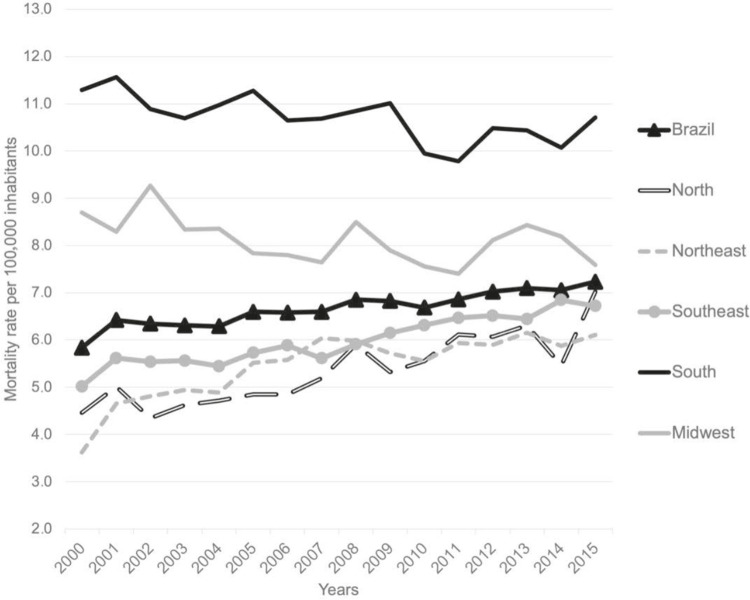
Trends of suicide mortality rates in adults aged 20 to 64 years of both genders, by Brazilian macroregions, 2000 to 2015.

Figure 1 illustrates trends in mortality rates for both genders. The upward trend in mortality for the whole of Brazil and the North, Northeast, and Southeast regions is clearly visible.

There was a tendency for suicide mortality to increase in each gender separately and for both genders taken together in the Northeast and Southeast regions and for the country as a whole, with statistically significant values (p<0.001). In the North region rates among males increased (0.26 a year) and also for both genders together (p<0.001); although the trend among females oscillated. Only the South and the Midwest regions had significant decreases in death by suicide rates. In both regions the overall population and the male subset trended downwards; however, the rate increased among the female population of the South region (0.04 a year) (p=0.002) ( Table 3 ).

**Table 3:** Annual trends in mortality by suicide among adults aged 20 to 64 years old, by gender and macroregions, from 2000 to 2015

Macroregions	Average annual change (95%CI)	Trend	p value	Adjusted R ^2^
North				
Male	0.26 (0.17 to 0.34)	Increase	**<0.001**	0.73
Female	0.01 (-0.00 to 0.04)	Oscillation	0.157	0.07
Both	0.13 (0.09 to 0.18)	Increase	**<0.001**	0.74
Northeast				
Male	0.21 (0.14 to 0.28)	Increase	**<0.001**	0.71
Female	0.03 (0.01 to 0.06)	Increase	**0.003**	0.44
Both	0.12 (0.08 to 0.16)	Increase	**<0.001**	0.70
Southeast				
Male	0.14 (0.11 to 0.18)	Increase	**<0.001**	0.84
Female	0.06 (0.05 to 0.07)	Increase	**<0.001**	0.90
Both	0.10 (0.08 to 0.12)	Increase	**<0.001**	0.90
South				
Male	-0.18 (-0.27 to -0.01)	Decrease	**<0.001**	0.61
Female	0.04(0.01 to 0.06)	Increase	**0.002**	0.45
Both	-0.07 (-0.11 to -0.02)	Decrease	**0.003**	0.45
Midwest				
Male	-0.09 (-0.18 to 0.00)	Decrease	**0.042**	0.21
Female	-0.00 (-0.04 to 0.02)	Oscillation	0.582	-0.04
Both	-0.05 (-0.10 to -0.00)	Decrease	**0.040**	0.21
Brazil				
Male	0.10 (0.07 to 0.12)	Increase	**<0.001**	0.81
Female	0.04 (0.03 to 0.05)	Increase	**<0.001**	0.82
Both	0.07 (0.05 to 0.08)	Increase	**<0.001**	0.88

95%CI = 95% confidence interval.

## Discussion

The absolute numbers of suicides increased over the years analyzed, following the gradual increase in the Brazilian population. The percentage of 1.72% of deaths found is slightly higher than the worldwide percentage in 2012, which was 1.4%. ^1^ Suicide mortality was higher among men in all regions and in all years presented, and a ratio of about 5 to 1 male to female deaths was observed, which is higher than the ratio of 3:1 found by Machado et al. ^11^ in a similar study analyzing data from 2000 to 2012.

The main methods chosen were hanging or strangulation, pesticide self-poisoning, and gunshot. In September 2017, the Ministry of Health’s department of surveillance published its first “epidemiological bulletin of suicide attempts and deaths”, containing an overview of data on the subject and associated prevention strategies. ^2^ This bulletin, evaluating a range of data from SIM from 2011 to 2016, shows that agricultural work is a profession with increased risk of suicide death, an association already shown in previous studies. ^2 , 14 , 15^ It also considers, among other causes, easy access to agrochemicals and pesticides, illegally sold at times or without good quality control by the regulatory agencies, emphasizing the importance of access to the resource for the correct purpose. ^7^ This also occurs with access to firearms, mostly from illegal trade, and access to controlled drugs. With profiles in which the method is difficult to control (for example, hanging/strangulation, common drugs, and jumping from high places), recognizing the profile of the person at risk is essential, as well as achieving immediate contact with and management of this individual by health professionals. ^11^

The same bulletin reports that, although the male population accounts for 80% of accomplished deaths by suicide, women try to commit suicide more frequently (69%) and tend to repeat the attempts 6% more than men. The population of suicide attempters analyzed in the bulletin preferred intoxication by drugs or other chemicals and harmful substances. The difference between attempts and consummated acts in the population can be related to more violent methods preferred by men (firearms, for example), rather than drugs or other substances, which are the first choice of women who attempt suicide and tend to be less lethal. ^2^

The North and Northeast regions showed significant upward trends. The trend in the Northeast region was the highest percentage increase among all the regions studied, although the rates in the North and Northeast remain the two lowest rates per 100,000 inhabitants in the study. It is of note that in poorly assisted regions, there is little or no incentive for the municipality/State to refine collection of data on mortality, since, at first sight, to do so would make the municipality or region in question have worse outcomes. Moreover, one must also consider the smaller scope of the healthcare network and the work of Psychosocial Care Centers (PCC) in the North region, which has the worst coverage of all 5 regions. ^16^

Furthermore, certain factors are considered “suicidogenic”, including local environmental and socioeconomic ills, such as drought, famine, rising violence in areas of vulnerability associated with use of drugs and alcohol, poor health conditions with consequent increase in infant and maternal mortality, incidence of psychiatric disorders without treatment, and labor exploitation – especially in rural areas. ^5 , 11^ There is consensus that the Human Development Index (HDI) is associated with violence – self-directed or otherwise. However, it is not possible to directly associate (in a cause-effect relationship) suicide rates with the HDI of the region, endorsing the greater importance of other risk factors in the outcome. ^17^ For example, Japan has an HDI of 0.89 and its coefficient of deaths by suicide was 21.4 for each 100,000 inhabitants in 2013, making it the country with the fifth highest absolute number of suicide deaths in the same year – 29,000. ^18 , 19^

Southern Brazil is a dilemma and a challenge. Although its trend was significantly downward, it remains the region with the highest rates of death from self-inflicted violence per 100,000 inhabitants. Between 1980 and 2006, suicide rates varied between 8.1 and 9.8%, reaching 10.4% between 1998 and 2000. Between 2010 and 2015, the South region accounted for 23% of self-harm deaths but only had 14% of the population. By comparison, 38% of suicides occurred in the Southeast region, where 42% of the country’s population lived. ^2^ Another study that investigated these deaths in the state of Rio Grande do Sul reported that most of them occur in rural areas or are associated with fishing activities and suggested that they may be associated with isolation, low professional recognition, low remuneration, subtropical climate (with higher incidence of seasonal depression), distance from large health referral centers, and, once more, contact with harmful chemicals such as pesticides, which have been repeatedly associated with the genesis of severe psychiatric disorders, such as depression, schizophrenia, and bipolar disorder, known to be the main risk factors for self-harm injury. ^7 , 14^

The downward temporal trends in the South and Midwest regions found in this study may be related to changes in social and demographic conditions experienced by these populations and to the fact that the reasons leading individuals to commit suicide vary between age groups, even among adults. Over the same period, the South and Midwest regions recorded a sharp drop in the Gini index (a coefficient that evaluates income concentration) and in unemployment and poverty. While, in common with the HDI, these coefficients cannot be associated as direct causes, these changes may partially explain the reduction in the exposure to socioeconomic risk factors for suicide. ^11^

Additionally, the progress in attempts to implement decentralization policies in healthcare, especially through the Family Health Strategy (ESF) program is known to have been successful in providing and improving access for the population to services and health professionals who, even those who do not receive specific training for suicide, can identify and manage patients who are at risk or have suicide ideation. This is especially true in the South and Southeast regions and, to a lesser extent, in the Midwest region. ^20^ Further studies are required for a more in-depth evaluation of regional characteristics and traits and to enable more accurate identification of associations.

This study collected data from the Ministry of Health’s Brazilian Mortality Database (SIM), implemented in 1975 and made compulsory in Brazil in 2000 by ordinance No. 474 of August 31, 2000, later substituted by ordinance No. 20, of October 3, 2003, which regulated data collection and flows and frequency of information sent to the Ministry of Health. It is recognized that mortality statistics are one of the best ways to evaluate health indicators and illness in the target population. However, since the SIM is a secondary database, it is directly influenced by the quality of completion of death certificates (regarding correctness and clarity of information) and the heterogeneous coverage in the different Brazilian regions. ^21^

The difficulty in collecting data leads to underreporting of deaths in some places, such as in rural areas, where undocumented burials occur. Without proper notification of the death to the organs, or of confusion factor of causa mortis, the intention to self-harm is not identified and the notification is submitted as accidental death (such as traffic accidents) or death from indeterminate causes. Detailed information on the victims such as race, place of residence (rural or urban), education, income or religion, as well as the microregional differences of incidence of death in each state/macroregion were not evaluated.

Few epidemiological studies have evaluated trends in suicide mortality in the Brazilian adult population. Existing data are scarce, and conclusions are imprecise and often express only the clinical view, aimed at the patient, and not at the population of which the individual is a member. Studying the epidemiological variables orients public health policies and improves understanding of the phenomenon of suicide, which, although a taboo subject, is very prevalent and little understood in all cultures.

## Conclusion

Mortality from intentionally inflicted self-harm increased in Brazil over recent years, especially in the male population from the North, Northeast, and Southeast regions. In the South and Midwest regions, there were significant downward trends in the male population and in the population as a whole (both sexes together). It is important to highlight that the national mortality coefficients hide very significant regional variations and are difficult to measure and also that Brazil is a country of continental proportions. Even regional analyses have significant intra-regional variations and further studies are needed to enable identification of more accurate associations (such as studies with age stratification). Economic, social, and cultural parameters are strongly linked to these differences and may be factors of increased risk or protection.
